# Inoculum production of *Phytophthora medicaginis* can be used to screen for partial resistance in chickpea genotypes

**DOI:** 10.3389/fpls.2023.1115417

**Published:** 2023-02-20

**Authors:** Sean L. Bithell, Andre Drenth, David Backhouse, Steve Harden, Kristy Hobson

**Affiliations:** ^1^ Plant Systems, New South Wales Department of Primary Industries, Tamworth, NSW, Australia; ^2^ Centre for Horticultural Science, University of Queensland, Brisbane, QLD, Australia; ^3^ School of Environmental and Rural Science, University of New England, Armidale, NSW, Australia

**Keywords:** root disease, phenotyping, tolerance, quantitative resistance, pathogen proliferation

## Abstract

*Phytophthora* root rot caused by *Phytophthora medicaginis* is an important disease of chickpeas (*Cicer arietinum*) in Australia with limited management options, increasing reliance on breeding for improved levels of genetic resistance. Resistance based on chickpea–*Cicer echinospermum* crosses is partial with a quantitative genetic basis provided by *C. echinospermum* and some disease tolerance traits originating from *C. arietinum* germplasm. Partial resistance is hypothesised to reduce pathogen proliferation, while tolerant germplasm may contribute some fitness traits, such as an ability to maintain yield despite pathogen proliferation. To test these hypotheses, we used *P. medicaginis* DNA concentrations in the soil as a parameter for pathogen proliferation and disease assessments on lines of two recombinant inbred populations of chickpea–*C. echinospermum* crosses to compare the reactions of selected recombinant inbred lines and parents. Our results showed reduced inoculum production in a *C. echinospermum* backcross parent relative to the *C. arietinum* variety Yorker. Recombinant inbred lines with consistently low levels of foliage symptoms had significantly lower levels of soil inoculum compared to lines with high levels of visible foliage symptoms. In a separate experiment, a set of superior recombinant inbred lines with consistently low levels of foliage symptoms was tested for soil inoculum reactions relative to control normalised yield loss. The in-crop *P. medicaginis* soil inoculum concentrations across genotypes were significantly and positively related to yield loss, indicating a partial resistance-tolerance spectrum. Disease incidence and the rankings for in-crop soil inoculum were correlated strongly to yield loss. These results indicate that soil inoculum reactions may be useful to identify genotypes with high levels of partial resistance.

## Introduction

*Phytophthora* root rot (PRR) of chickpea (*Cicer arietinum*), caused by the Oomycete, *Phytophthora medicaginis*, is an important root disease of chickpea crops in the north-eastern Australian grain belt (Singh et al., 1994; Salam et al., 2011). For chickpea, similar to PRR of soybean, treatment of the seed with metalaxyl provides initial control during crop establishment, but protection across the whole growing season using cost-effective chemicals is not available (Dorrance and McClure, 2001). Absence of effective control methods has led to a focus on breeding for improved levels of resistance to provide a genetic solution to control PRR in chickpeas (Singh et al., 1994).

There are various types of resistance to plant pathogens that have different genetic basis. In this study, we examined partial resistance, which we define as resistance that confers reduced pathogen development, propagation, and spread of a disease in a plant population with a quantitative (non-major gene) genetic basis (Pariaud et al., 2009; St Clair, 2010) (Glossary Box). The term tolerance has also been used widely to refer to the performance of a genotype under disease pressure in the field, especially the ability to maintain yield in the presence of infection, although there have been considerable contradictions in its use and interpretation (Simms and Triplett, 1994; Erwin and Ribeiro, 1996; Pagan and Garcia-Arenal, 2020) (Glossary Box). From a general perspective of cause and effect between plant and pathogen, resistance is considered the effect of the plant on the pathogen, whereas tolerance is considered the effect of the pathogen on the plant. To discriminate among genotypes with partial resistance or tolerant phenotypes, it is necessary to compare the fitness or productivity of genotypes under the same levels of pathogen colonisation (Schafer, 1971; Pagan and Garcia-Arenal, 2020). Pagan and Garcia-Arenal (2020), when reviewing this area, observed that it is technically difficult to ensure the same level of pathogen colonisation even in non-field-based phenotyping systems but that quantifying the amount of disease or inoculum in the relevant infected tissue provided an effective method of comparing reactions and that fitness could be normalised against control treatments.

## Glossary box

*Partial resistance*: the resistance that confers reduced pathogen development, propagation, and spread of a disease in a plant population with a quantitative (non-major gene) genetic basis.

*Tolerance*: the performance of a genotype under disease pressure in the field, especially the ability to maintain yield in the presence of infection.

The evaluation of material to provide improved resistance to *P. medicaginis* in chickpeas is ongoing. Early field and glasshouse screening studies of chickpea germplasm identified lines with improved survival times, but findings demonstrated inconsistent responses between field and glasshouse reactions (Dale and Irwin, 1991). *C. arietinum*-based chickpea varieties, such as var. Yorker, were released with a moderately resistant rating for PRR based on foliage symptom assessments (Knights et al., 2009). Although Yorker has a level of improved resistance, field evaluations showed that this resistance was not effective under conditions of high disease pressure and in seasons conducive to PRR development (Bithell et al., 2021). Furthermore, var. Yorker produced high *P. medicaginis* inoculum concentrations in soil at the end of the season even under moderately conducive conditions (Bithell et al., 2021). The inoculum and yield results for var. Yorker were indicative of a tolerance-type reaction or weak partial resistance. The absence of effective resistance sources in *C. arietinum* to *P. medicaginis* led to the evaluation of alternative resistance sources including wild relatives of chickpeas (Singh et al., 1994; Li et al., 2015). Among a range of wild relatives of chickpeas, *Cicer echinospermum* accessions provided the longest survival times in the presence of PRR infection; partial resistance was demonstrated by an absence of absolute resistance with the *C. echinospermum* accessions eventually dying from PRR (Knights et al., 2008). Resistance from *C. echinospermum* was successfully transferred to the progeny of crosses with chickpeas, and loci were identified for a complex quantitative genetic basis to the partial resistance (Knights et al., 2008; Amalraj et al., 2019).

Consistent selection or phenotyping across seasons, in systems with partial resistance, can be challenging, as the expression of resistance is highly dependent on the prevailing environmental conditions. Genotype-by-environment interactions involving partial resistance may be due to differing resistance thresholds among genotypes across a pathogen density gradient resulting in differing infection intensities among genotypes (Price et al., 2004). Recombinant inbred lines (RILs) of two chickpea–*C. echinospermum* populations provided a number of major quantitative trait loci (QTL) for resistance to *P. medicaginis* that showed negligible interactions for environments, while other resistance QTL showed strong environmental interactions (Amalraj et al., 2019). In some pathosystems, partial resistance may occur in combination with tolerance traits (Poland et al., 2009; Mikaberidze and McDonald, 2020). However, determination of the relative contribution of partial resistance and tolerance traits to disease reaction outcomes in variable field environments is difficult (Simms and Triplett, 1994; Masini et al., 2019; Pagan and Garcia-Arenal, 2020). The selection of material containing both partial resistance and disease tolerance traits was shown in one case to provide an inadvertent selection of tolerance over resistance traits (Mikaberidze and McDonald, 2020).

Current Australian chickpea breeding objectives involve finding the most beneficial combination of alleles to achieve high levels of disease resistance with high grain yield and quality. We sought to determine if selection for high-yielding lines under PRR disease pressure also selects material with high levels of partial resistance. It was also important to determine if changes in the amount of *P. medicaginis* inoculum and levels of disease severity are linked to other traits that may be more easily measured in a high-throughput breeding program to improve the selection process for high-yielding partially resistant material.

We specifically sought to determine if

1. inoculum production differs among RIL and RIL parents with differing levels of PRR resistance,
2. inoculum production differs relative to normalised yield loss among RIL lines selected for low levels of PRR development, and
3. there are disease or plant parameters that relate to *P. medicaginis* inoculum production values.

An in-depth understanding of disease assessment methods, inoculum responses, and trait composition is required to maximise sustainable yield and achieve genetic gain for resistance in chickpeas against *P. medicaginis* in breeding programs.

## Materials and methods

To test our hypotheses, we used two RIL populations of a *C. echinospermum* backcross*susceptible population and a *C. echinospermum* backcross*tolerant population. The populations were phenotyped for their levels of PRR resistance in field experiments, including grain yield and *P. medicaginis* soil inoculum development at the harvest of selected RIL material.

### RIL development and seed sources

A moderately PRR-resistant breeding line, 04067-81-2-1-1(B), which is a *C. arietinum* backcross *C. echinospermum* (Howzat/ILWC 245//99039-1013), was used to develop two F6-derived RIL populations by the National Chickpea Breeding Program based at the New South Wales Department of Primary Industries, Tamworth. The first population (D09008) was a cross of 04067-81-2-1-1(B) and an Australian PRR-susceptible chickpea variety, Rupali (pedigree: FLIP84-15C/ICCV88516//Amethyst); this is hereafter referred to as the BC*susceptible RIL population. The second population (D09024) was a cross of 04067-81-2-1-1(B) and an Australian desi chickpea variety, Yorker (pedigree: 8507-28H/946-31); this is hereafter referred to as the BC*tolerant RIL population. Yorker was released as a PRR moderately resistant chickpea variety, with resistance ratings based on foliage symptoms (Knights et al., 2009).

### Isolates and inoculum production

*P. medicaginis* is a homothallic species. Ten isolates of *P. medicaginis* were used (as a mixture) in all experiments, storage, and isolate culturing as described in Bithell et al. (2022). Prior to inoculum production, each isolate was passaged through plants in a glasshouse using the very susceptible chickpea variety Sonali to ensure pathogenicity. With the use of low-strength V8 media (100 ml of V8 juice, 10 g of agar, 2.5 g of calcium carbonate, and 900 ml of Milli-Q water), an oospore suspension was prepared by macerating cultures with a hand-held Braun 600W blender and then added to flooded (Milli-Q water) cups of seedlings in potting mix, which were then drained after 48 h. After the observation of wilting, chlorosis, and canker development on the seedlings, stem tissue at the margin of the canker was used to re-isolate the pathogen on corn meal agar. Cultures were hyphal tipped and then grown on low-strength V8 media. Subcultures of these freshly passaged isolates were used to produce 90-mm-diameter Petri dish cultures of each isolate, which were grown in the dark at 21°C–23°C for at least 6 weeks prior to mixing with Milli-Q water (10% V/V) and macerating using a hand-held Braun 600W blender for approximately 3 min. Average oospore concentrations for each isolate were determined using counts under a 20 * 50-mm coverslip to prepare inoculum mixtures containing equal oospore concentrations.

### RIL population disease status and phenotype selection

The two RIL populations were phenotyped for the severity of PRR development in inoculated field experiments in order to select RILs with high and low disease phenotypes.

*Field experiments*: The BC*susceptible RIL (n = 181) and the BC*tolerant RIL (n = 165) population were sown on 18 and 19 June 2014 in separate experiments at Hermitage Research Facility, Queensland (−28.204908 S, 152.102689 E) in 2014. The methods used for the RIL field experiments are described by Amalraj et al. (2019). Briefly, the plots were sown with a four-row seeder with separate in-furrow delivery of in-solution *Mesorhizobium ciceri* rhizobia inoculant and the 10 isolate mixture of *P. medicaginis* at sowing at a concentration of ~1,500 oospores/seed. Each plot had 20 seeds per single 1.2-m row plot. The experiments had a randomised block design with four replicates. Check varieties covering a resistance spectrum were supra-replicated on block and sub-block basis. The soil type at the Hermitage site was a deep, self-mulching, black vertosol (Thomson et al., 2007). No in-crop irrigation was applied, and 97 mm of in-crop rainfall was received during the field experiments.

*Establishment and disease assessments*: The number of seedlings in each plot was counted 48 days after sowing (DAS) to determine establishment. A minimum of three disease assessments were then made; the first assessment was performed when early disease symptoms were evident in susceptible check varieties (85 DAS, pre-flowering 12–14 nodes), the second assessment was made mid-season (118 DAS, immature pods present), and the final assessment (135 DAS) occurred at the beginning of pod maturity. At each disease assessment, separate counts of the number of chlorotic, dead, and total number of plants were made. Late-season assessments were carried out before widespread plant senescence had occurred. At the final assessment, dead plants were categorised into development categories as having produced no pods (died as seedlings prior to flowering) or as podded, and counts of each category were made. At this assessment, counts were also made of the number of chlorotic, senescent, and healthy non-senesced plants.

*Selected RIL disease phenotype groups*: To select RILs with high and low disease phenotypes, the proportion of plants that had died at the 135 DAS assessment timing was used as the criterion. From each RIL population, six lines were randomly selected as low disease lines using a random number function in Excel (Microsoft Office Standard, 2016) on the basis of having no plant death. Six high-disease RILs were randomly selected from each RIL population based on more than 30% plant death for the BC*susceptible RIL and greater than 10% plant death for the BC*tolerant RIL.

### High and low disease RIL inoculum relationship

The soil beneath the 24 selected RIL and parents of the two RIL populations was sampled to evaluate *P. medicaginis* inoculum concentrations across different disease phenotypes. Each plot of the selected RIL in the above experiments was soil sampled 145 days after sowing (DAS), by taking four separate 45-mm-diameter 100-mm-depth soil cores, each 250 mm apart; two were collected from either side of each row, approximately 20 mm from the closest stem base, placed in bags and dried at 40°C for 72 h. A 500-g sub-sample was then sent to the Root Disease Testing Service at the South Australian Research and Development Institute (Adelaide, Australia) to quantify the *P. medicaginis* soil DNA concentration as described by Bithell et al. (2021).

### Superior RIL yield loss and inoculum production

We compared selected RIL to determine if inoculum production relative to normalised yield loss differed. We used a set of eight superior RIL, which had provided consistently low disease reactions (BC*susceptible (n = 3), the maximum proportion of dead plants from back-transformed logits for the three selected RIL, range 0.015 to 0.049; and BC*tolerant (n = 5), the maximum proportion of dead plants from back-transformed logits for the five selected RIL, 0 to 0.018) across three phenotyping experiments per population (Amalraj et al., 2019). A four-row plot (each 15 m^2^) experiment was conducted at Hermitage, as a randomised complete block design with three replicates. The experiment was sown on 27 June 2017 with an in-furrow delivery of in-solution *M. ciceri* rhizobia. All seeds had a seed treatment of 360 g/L of thiram and 200 g/L of thiabendazole. There was an uninoculated control (−Pm) treatment, where the seed was also treated with metalaxyl (350 g/L of metalaxyl-M, 75 ml/100 kg seeds), and the plots received metalaxyl soil drenches [Ridomil Gold 480 SL (480 g/L of metalaxyl-M, 0.4 mL/L water/m of row)] at six weekly intervals after sowing. There was a *P. medicaginis* inoculated (+Pm) treatment, where an in-furrow application of a solution of *P. medicaginis* oospores and mycelium was applied at sowing as described for the earlier experiment. When the −Pm treatment received metalaxyl soil drenches, the +Pm treatment received water drenches equivalent to the metalaxyl application (1 L water/m of row).

Plots were sown at calculated seed densities to achieve a target population of 35 plants/m^2^. Around each experimental plot, four-row buffer plots of metalaxyl-treated var. Yorker seeds were planted to prevent the movement of *P. medicaginis* between treatments. Supplementary irrigation of 31 mm was applied with dripper tape (T-tape, Rivulas Irrigation) over a 5-day period starting 35 DAS. There was 137 mm of in-crop rainfall during this field experiment. Disease assessments were carried out on the middle two rows of each plot. The number of chlorotic and/or dead plants was counted in each plot at approximately 6-week intervals. Plant heights were recorded by measuring two plants per plot at physiological maturity (141 DAS). The proportional area of early senescence was also assessed for each plot on this date. The middle two rows of each plot were machine harvested at 169 DAS to determine grain yield.

To determine inoculum production in-crop (140 DAS) and postharvest (170 DAS), five soil cores were collected from each of the middle two rows of each plot, using 45-mm-diameter 100-mm-depth soil cores collected approximately 20 mm from the closest stem base. The pooled cores from each plot were dried at 40°C for 72 h. All plots were sampled at 170 DAS, but only the BC*tolerant RIL and parents (n = 7) were sampled at 140 DAS. After being dried, a 500-g sub-sample was collected using the level surface sub-sampling method described by Schroth (2003) and sent for *P. medicaginis* DNA concentration analysis as previously described.

### Design and analyses

All experiment layouts were designed using DiGGer ver. 1.0.2 (Coombes, 2016). The two RIL population experiments that included check varieties were supra-replicated on block and sub-block basis. Residuals were examined, and if necessary, data were appropriately transformed to meet requirements for residuals to be normally distributed. Residual degrees of freedom are presented for each analysis.

*Hermitage RIL population experiments*: For the two whole RIL population experiments, RIL with complete data across all replicates was selected for analysis. This provided 173 RIL for the BC*susceptible population and 164 RIL for the BC*tolerant population. Analysis of the proportion of dead seedlings (dead with no pods), dead podded plants, chlorotic or senescent podded plants, non-symptomatic podded plants, and all plants with pods from the final disease assessment was made with a generalized linear mixed model (GLMM) with a binominal distribution logit link and the Wald test. The back-transformed logit values for each RIL were then used for whole-population comparisons among disease and development parameters. For the RIL from the high and low disease classes, ANOVA with RIL nested within the disease class was used to compare *P. medicaginis* DNA concentrations and disease parameters.

*Superior RIL yield loss and inoculum production*: A GLMM binominal distribution logit link and the Wald test was used for the analysis of the proportion of dead and chlorotic plants. Grain yield and height reduction data were normalised relative to the metalaxyl-protected control treatment as outlined for the determination of point tolerance responses (Pagan and Garcia-Arenal, 2020). After the evaluation of a range of models, regression with an exponential function was used to assess the relationship between the proportion of infected plants and normalised yield, and linear regression was used to assess the relationship between other parameters.

All statistical analyses were carried out with GenStat 19th edition (Anon, 2018).

## Results

### RIL population disease status at maturity and phenotype selection

**Figure 1:**
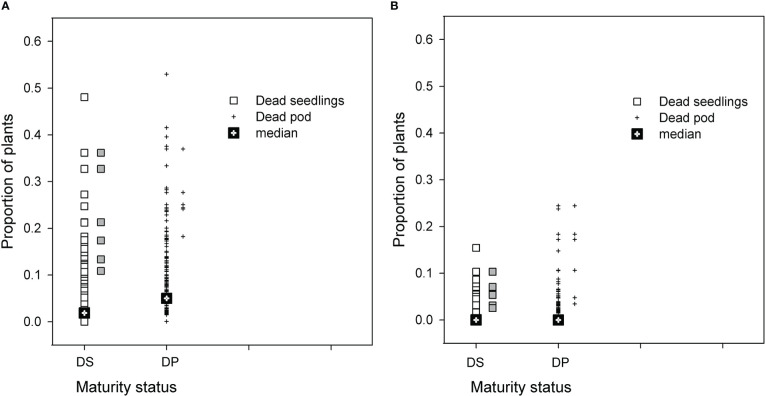
Results for two recombinant line (RIL) populations, **(A)** *Cicer echinospermum* backcross*susceptible (BC*susceptible) and **(B)** *C. echinospermum* backcross*tolerant (BC*tolerant), in two *Phytophthora medicaginis* inoculated single-row experiments, for proportions of plants dead at the final assessment with a development status categorised as dead seedlings (DS, □) or dead podded plants (DP, +). Median values for each category presented and symbols in grey to the right of each population plot are the six selected high disease RIL that were postharvest soil sampled.

The distribution of PRR disease of RIL in both populations was used to select groups of RIL with high and low disease phenotypes. Seasonal conditions in 2014 were not conducive to high levels of PRR development, but the BC*susceptible RIL had close to a proportion of 0.5 of plants dying as either seedlings or podded plants (**Figure 1A**). Recombinant inbred lines with proportional total mortality (dead seedling plus dead podded plants) values ranging from 0.32 to 0.62 were selected as the high disease group. For the BC*tolerant RIL, few lines had a greater proportion than 0.20 mortality in any development category (**Figure 1B**). Lines selected from this population for the high disease group had total mortality proportional values ranging from 0.11 to 0.31. Comparison of the maturity status at the final assessment for the selected RIL between the two populations showed that the selected BC*tolerant RIL had three lines with a higher proportion of dead podded plants than dead seedlings. In contrast for the selected BC*susceptible RIL, the proportion of dead seedlings was as high as dead podded plants.

### High and low disease RIL inoculum relationships

This analysis was completed to test for differences in *P. medicaginis* inoculum between the high and low disease phenotypes in each of the contrasting RIL populations.

**Figure 2:**
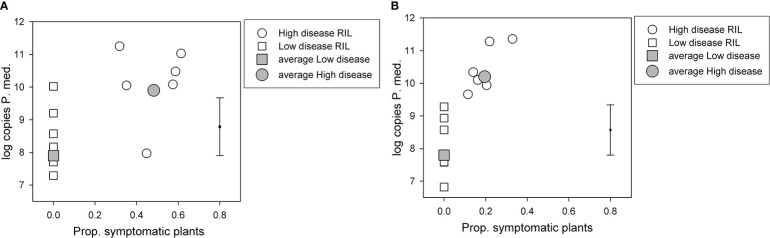
Results for selected recombinant inbred lines (RILs) in two *Phytophthora medicaginis* inoculated single-row experiments, with six low disease and six high disease lines from **(A)** *Cicer echinospermum* backcross*susceptible (BC*susceptible) and **(B)** *C. echinospermum* backcross*tolerant (BC*tolerant) populations, with the proportion of plants with foliage symptoms (chlorotic plus dead) plotted against the log-transformed postharvest soil *P. medicaginis* (P.med) DNA concentrations (number of sequence copies/g soil) for individual RIL and high and low disease group averages. Error bar shows LSD (p < 0.05) for disease group analysis. LSD, least significant difference.

For the BC*susceptible RIL population, there was a significant (p < 0.05, df = 33, least significant difference (LSD) = 1.77) difference in log-transformed soil *P. medicaginis* DNA values among the two disease groups, the high disease group had a value of 9.9, and the low disease group had a value of 7.9 (**Figure 2A**). However, log-transformed soil *P. medicaginis* DNA values did not differ significantly (p > 0.05) between the two parents, 04067-81-2-1-1(B) (6.0) and Rupali (5.9). 04067-81-2-1-1(B) and Rupali differed significantly (chi probability = 0.003) in the proportion of non-symptomatic plants that produced pods, with respective back-transformed logit proportions of 0.94 and 0.51. The *C. arietinum* parent of the BC*tolerant population, var. Yorker, was included as a check in this BC*susceptible RIL population experiment and provided a high (11.1) postharvest *P. medicaginis* DNA value.

For the BC*tolerant RIL population, there was also a significant (p < 0.05, df = 33, LSD = 1.54) difference in log-transformed soil *P. medicaginis* DNA values among the two disease groups, the high disease group had a value of 10.2 and the low disease group a value of 7.8 (**Figure 2B**). For the parents of this population, log-transformed soil *P. medicaginis* DNA values differed significantly (p < 0.05, residual df = 3, LSD = 1.81) between 04067-81-2-1-1(B) (7.4) and var. Yorker (10.0). 04067-81-2-1-1(B) and Yorker differed significantly (chi probability < 0.001) in the proportion of non-symptomatic plants that produced pods, with respective back-transformed logit proportions of 1.00 and 0.851.

### Superior RIL yield loss and inoculum production

This experiment tested whether inoculum production relative to normalised yield loss differed among three RIL from the BC*susceptible RIL population and five RIL from the BC*tolerant population selected for superior performance and two parents. In addition, we sought to identify which disease or plant parameters may relate to genotype inoculum production values.

There was a significant range in the proportion of PRR symptomatic (dead plus chlorotic) plants, especially among the parents of the BC*tolerant RIL population where var. Yorker had a high symptomatic proportion (0.76), while the other parent 04067-81-2-1-1(B) and five other RIL had values below 0.03 (**Table 1**). The RIL with the highest symptomatic proportion of 0.13 was from the BC*susceptible population.

**Table 1:** Superior recombinant inbred line (RIL) disease (proportion of symptomatic plants), early senescence (Early Sen.) results for Phytophthora medicaginis (+Pm) inoculated RIL and yield results from control (-Pm) and inoculated RIL from the Cicer echinospermum backcross*susceptible (BC*sus.) and C. echinospermum backcross*tolerant (BCxtol.) populations and two RIL parents.

		Prop. Symp.	Early Sen.	Grain, kg/ha	
Population	Genotype	+Pm	% area	−Pm	+Pm
	04067-81-2-1-1(B)	0.02	13.3	2,885	2,148
BC*sus.	D09008B>F6RIL>046	0.01	47.5	3,354	2,117
BC*sus.	D09008C>F6RIL>007	0.06	32.1	3,771	2,796
BC*sus.	D09008D>F6RIL>016	0.13	50.8	3,075	1,990
BC^x^tol.	D09024B>F6RIL>020	0.08	35.0	3,403	1,639
BC^x^tol.	D09024B>F6RIL>030	0.02	10.0	2,456	1,948
BC^x^tol.	D09024B>F6RIL>040	0.01	13.3	3,044	3,093
BC^x^tol.	D09024C>F6RIL>010	0.04	25.4	2,886	2,346
BC^x^tol.	D09024D>F6RIL>028	0.01	20.0	3,483	3,093
	Yorker	0.76	77.5	3,135	527
Wald/LSD		122.1^W^	24.27	1,014.7		

Wald statistic presented for the proportion of symptomatic (Prop. Symp.) plants, Wald tests, ^W^p < 0.001 . Least significant difference (LSD) presented for area of early senescence (Early Sen.), and grain yields for the control (−Pm) and +Pm treatments in 2017 yield loss experiment.

The area of early senescence differed among *Phytophthora* treatments (p < 0.001, −Pm 16.2%, +Pm 46.7%, LSD = 10.86) and among genotypes (p < 0.001), but there was no significant interaction. For grain production, there was a significant interaction (p < 0.05) where four RIL and the parent, 04067-81-2-1-1(B), did not have a significant reduction in yield in the +Pm treatment relative to −Pm. In addition, for the +Pm treatment, two RILs had higher yields than three other RILs, including two from the same BC*tolerant population. The four genotypes that had significant reductions in yield were also the only genotypes to have early senescence values of 35% or greater.

Comparison of genotype traits for relationships between disease, plant height, yield, in-crop, and postharvest inoculum production parameters was based on the use of normalised results. For genotype effects (df = 29), there were significant differences for normalised plant heights (p < 0.001), normalised yield losses (p < 0.05), and postharvest soil *P. medicaginis* inoculum concentrations (p < 0.05).

**Figure 3:**
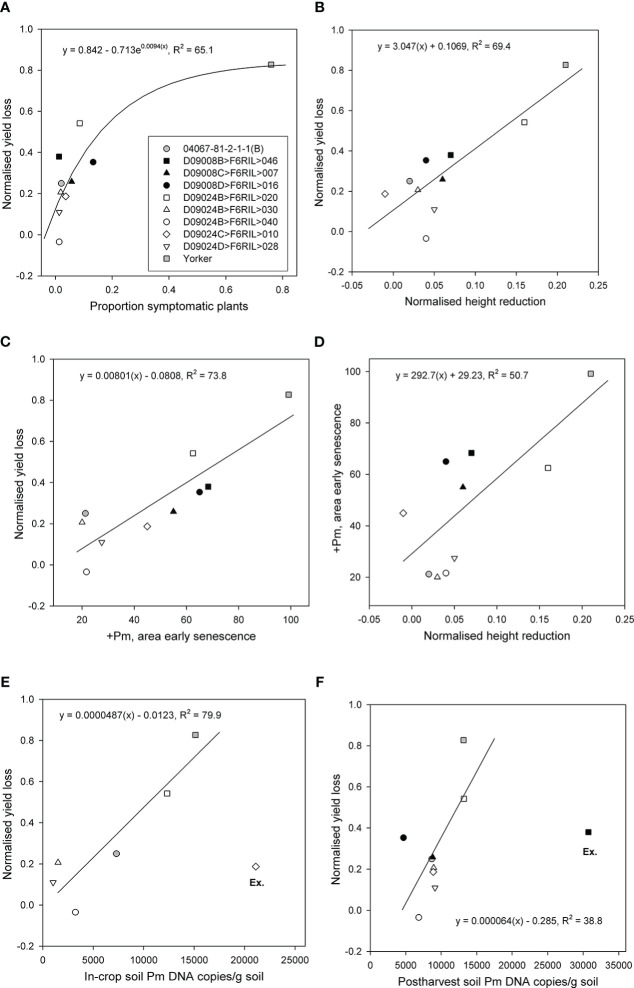
Relationships for eight superior recombinant inbred lines (RIL) from two populations (*Cicer echinospermum* backcross*susceptible (D09008) and *C. echinospermum* backcross*tolerant (D09024)) and two parents for plots of **(A)** back-transformed proportion of symptomatic plants *vs.* normalised yield loss, **(B)** normalised height reduction *vs.* normalised yield loss, **(C)** area of early senescence *vs.* normalised yield loss, **(D)** normalised height reduction *vs.* area of early senescence, **(E)** in-crop *Phytophthora medicaginis* (Pm) DNA concentrations *vs.* normalised yield loss, and **(F)** postharvest *P. medicaginis* DNA concentrations *vs.* normalised yield loss. Fitted regression lines and equations are presented. For **(E, F)**, one RIL was excluded (Ex.) from each regression.

The proportions of symptomatic plants, normalised height reduction, and area of early senescence were significantly and positively related to normalised yield loss. The proportion of symptomatic plants accounted for 65% (p < 0.05, df = 7) of the variance in normalised yield loss across the RIL and parents (**Figure 3A**). Normalised height reduction (p < 0.01, R^2^ = 69.4) and the area of early senescence in the +Pm treatment (p < 0.001, R^2^ = 73.8) both accounted for a substantial proportion of the variance in normalised yield loss (**Figures 3B,C**), although RIL with the least height reduction did not have the lowest yield loss and vice versa. Normalised height reduction also accounted for approximately half of the variance in the area of early senescence in the +Pm treatment (p < 0.05, R^2^ = 50.7) (**Figure 3D**).

In-crop soil *P. medicaginis* DNA concentrations were assessed for the BC*tolerant population RIL and parents. For comparisons with proportional yield loss, one RIL D09024C>F6RIL>010 had the highest in-crop *P. medicaginis* DNA value but less than 20% proportional yield loss. When that particular RIL was excluded as an outlier, the in-crop soil *P. medicaginis* DNA concentrations accounted for a large (p < 0.05, R^2^ = 79.7) proportion of the variance in proportional yield loss. With no exclusions, there was no significant (p > 0.05) relationship (**Figure 3E**). Postharvest soil *P. medicaginis* DNA concentrations were assessed for all entries, but a different RIL (D09008B>F6RIL>046) provided high *P. medicaginis* DNA values but mid-range proportional yield loss values. When that particular RIL was excluded, the postharvest soil *P. medicaginis* DNA concentrations accounted for a moderate (p < 0.05, R^2^ = 38.8) proportion of the variance in proportional yield loss (**Figure 3F**).

Comparison of RIL performance across multiple parameters that identified three of the five RILs from the BC*tolerant population provided low-range normalised height reductions and early senescence values, in addition to consistently low in-crop and postharvest *P. medicaginis* DNA concentration results (**Figure 3**). For the six genotypes used in the in-crop inoculum regression, genotype in-crop inoculum values were significantly (p < 0.05) correlated with both normalised height reduction (0.883) and early senescence (0.892). However, the rankings of genotypes with low in-crop inoculum values against normalised yield loss more closely matched the rankings of genotypes with low early senescence against normalised yield loss than height reduction-based genotype rankings. The genotype rankings for normalised yield relationships with the proportion of symptomatic plants, area of early senescence, and in-crop inoculum showed that two RILs (D09024D>F6RIL>028, D09024B>F6RIL>040) were consistently at the lower end of these three normalised yield loss relationships.

## Discussion

We evaluated *P. medicaginis* soil inoculum production of RIL from two chickpea–*C. echinospermum* populations. For groups of RIL with differing levels of foliage symptoms, *P. medicaginis* inoculum concentrations were higher for high disease phenotypes than low disease phenotypes in both RIL populations. Analysis of the soil inoculum reactions relative to control normalised yield loss for a set of RIL with superior PRR resistance required the exclusion of some RILs due to an apparent uneven distribution of inoculum in the field experiment. However, in-crop *P. medicaginis* soil inoculum concentrations were significantly related to normalised yield loss and indicated a putative partial resistance-tolerance spectrum.

Differences between the two disease phenotype categories in inoculum concentrations of RIL were related to the susceptibility and resistance of the RIL parents. The differences between high and low disease phenotypes for RIL from the BC*tolerant population could be related to differences in inoculum production values of their parents, where var. Yorker had significantly higher *P. medicaginis* soil inoculum concentrations than the backcross, 04067-81-2-1-1(B). Higher soil *P. medicaginis* inoculum production with var. Yorker was also confirmed in the BC*susceptible population experiment. The var. Yorker in-crop soil *P. medicaginis* inoculum concentrations were more than double those of 04067-81-2-1-1(B) in a separate study where genotypes were inoculated with the same equal oospore-based isolate mixture used in this study (Bithell et al., 2022). Furthermore, when soil *P. medicaginis* inoculum concentrations are expressed relative to root weight, var. Yorker had more than a 15-fold higher pathogen DNA concentration than 04067-81-2-1-1(B). Together, these results provide consistent evidence that the *C. echinospermum* backcross 04067-81-2-1-1(B) produced significantly less *P. medicaginis* inoculum than the moderately susceptible *C. arietinum* variety var. Yorker under field conditions. The lower inoculum production of 04067-81-2-1-1(B) could be attributed to the effects of partial resistance resulting in less extensive pathogen proliferation, as reported with other partially resistant material in similar pathosystems (Dorrance et al., 2001; Mideros et al., 2007). It follows that the *P. medicaginis* inoculum concentrations of the BC*tolerant RIL with high disease phenotypes were also similar to var. Yorker with elevated soil DNA values.

There was also evidence for particular RIL parents with differing PRR resistance to have similar *P. medicaginis* soil concentrations. We showed there was no difference in soil *P. medicaginis* concentrations in the soil among the parents of the BC*susceptible RIL population, viz., the very susceptible rated variety Rupali and 04067-81-2-1-1(B). In a prior study, *P. medicaginis* inoculum concentrations in the soil for another very susceptible PRR-rated variety, var. Sonali, showed a large decline in DNA concentrations from when peak disease and premature plant death occurred early in the growing season (Bithell et al., 2021). For this current study, of the three parents in the RIL population experiments, the very susceptible var. Rupali had the highest proportion of symptomatic plants (~49%) of all RIL parents. The high disease incidence included premature plant death for Rupali, which may be expected to have contributed to inoculum decline occurring prior to the inoculum sampling at the end of the growing season However, in contrast to results for the BC*tolerant RIL, five high disease BC*susceptible RILs had high inoculum concentrations, indicating that post-peak disease inoculum decline was not a trait of high disease BC*susceptible RIL.

Pathogen proliferation and normalised fitness assessments are key methods for the separation of genotypes with putative partial resistance or tolerance traits. It has been established in a number of soil-borne pathosystems, including those with oomycetes, that inoculum is often clustered in foci as opposed to a homogenous distribution (Campbell and Noe, 2003; Moussart et al., 2009). We demonstrated a large and significant range of normalised yield loss reactions to PRR infection among parents and eight chickpea–*C. echinospermum* RIL. However, we also identified issues probably related to the uneven distribution of *P. medicaginis* inoculum in the experiment. The uneven distribution of *P. medicaginis* inoculum may have contributed to variable inoculum results for some genotypes in the large plot yield loss experiment, as these also had a lower soil sampling intensity than the single-row plots in the first two experiments. It was necessary to exclude two genotypes from the analyses due to inconsistent soil inoculum values; however, analyses of the available data provided a number of useful findings. It will be important to identify *P. medicaginis*-free areas for experiments or to carry out plot-level sampling prior to sowing in order to reduce potential spatial variation in *P. medicaginis* populations across experimental sites. Issues of sample variability effects on cereal root pathogen detection can be addressed through the use of larger (250 g) sub-samples and the separation and grinding of soil organic matter prior to sub-sampling (Herdina and Roget, 2000). Prior to root decomposition, the highest concentrations of *P. medicaginis* inoculum are in root tissues. To reduce *P. medicaginis* detection variability, it may be appropriate to evaluate more samples and larger sample sizes (through a greater coring intensity per plot) and then separate and grind the organic matter in soil samples for re-inclusion with soil prior to DNA analyses.

Results for var. Yorker demonstrated a tolerance-type reaction due to substantial pathogen proliferation in association with the collapse of yield under high disease pressure. Both of these aspects were identified previously for var. Yorker but not in relation to control normalised yield loss (Bithell et al., 2021). Similar observations have been made in related pathosystems such as the PRR of soybean for genotypes with low levels of partial resistance, where under high inoculum and favourable environmental conditions, substantial yield losses could not be prevented from occurring (Dorrance et al., 2003). Based on our findings for var. Yorker, we determined that var. Yorker was a tolerant genotype through the demonstration of reduced fitness or productivity relative to pathogen proliferation (Pagan and Garcia-Arenal, 2020). From the identification of var. Yorker as tolerant, we were then able to interpret the positions of the remaining genotypes in the inoculum–yield loss relationship as representing a partial resistance-tolerance spectrum. The in-crop inoculum–yield loss relationship identified the BC*tolerant RILs that had the lowest in-crop and postharvest *P. medicaginis* concentrations and low PRR yield loss values. These RILs were therefore interpreted to have the highest levels of partial resistance. A number of QTL associated with both Yorker and the *C. echinospermum* backcross 04067-81-2-1-1(B) from analysis of foliage symptoms caused by PRR were assumed to represent partial resistance traits (Amalraj et al., 2019). The research presented in this current study provides evidence that some QTL associated with var. Yorker may be linked to tolerance traits. These findings reinforce the need for an improved understanding of the genetic basis of partial resistance and tolerance traits in *C. echinospermum* derivatives. In addition, it may be possible to determine the effect of crossing on QTL pyramiding and trait composition in *C. echinospermum* derivatives. Of relevance to these goals is research on crown rot of wheat caused by *Fusarium pseudograminearum*, which has shown the capability to separately identify partial resistance from tolerance QTL for a number of traits (Rahman et al., 2021).

There was evidence for both pre-flowering and post-flowering PRR disease effects on normalised yield loss. In studies of PRR-affected soybean varieties with differing levels of partial resistance, the ratio of plants producing grain in yield component analysis was the most critical factor contributing to yield loss (Tooley and Grau, 1984). Chickpea yield is a highly heritable trait, with overall yields of desi chickpeas in semi-arid environments largely dependent on two yield components: pods per unit area and seed weight (Gan et al., 2003; Ali et al., 2009). Pre-flowering PRR disease reduces the potential number of chickpea pods, but in our yield loss experiment, there were minor to nil foliage symptoms or plant death across all RIL in pre-flowering assessments (data not presented). The importance of the final proportion of symptomatic plants on normalised yield loss may then be linked to post-flowering effects. In contrast, we found that root disease effects on genotype vegetative growth (normalised height reduction) accounted for a substantial proportion of the variation in normalised yield loss, but only one RIL genotype had more than a 10% PRR incidence based on foliage symptoms prior to physiological maturity when height measurements were taken. The reductions in height had been induced in the preceding months when most of the foliage was non-symptomatic. For soybean, reductions in plant height from PRR infection were correlated with final disease incidence and severity but were not a predictor of yield loss (Tooley and Grau, 1984). Pre-flowering destructive root disease assessments would be required to determine the relationship between chickpea height reduction and disease severity effects on grain yield.

It was notable that relationships among parameters varied in some respects. The positive but non-linear relationship between proportional infection and normalised yield loss indicated that the extent of proportional yield loss was lower at the high level of infection for var. Yorker. However, linear relationships that included var. Yorker were observed between the parameters normalised height reduction, area of early senescence, and in-crop inoculum production. The non-linear normalised yield to disease incidence relationship may be linked to increased seed production from surviving plants in plots due to higher levels of pathogen resistance, reduced interplant completion, or a combination of these factors as reported for *P. medicaginis*- and *Phytophthora sojae*-inoculated chickpea and soybeans plants, respectively, in field experiments (Wilcox and St Martin, 1998; Miranda, 2019).

Alternative methods of identifying material with low levels of pathogen proliferation may be useful. Comparison of genotype rankings among parameters related to normalised yield loss showed the proportion of symptomatic plants and area of early senescence as the parameters that provided genotype rankings that most closely matched those at the base of in-crop inoculum–normalised yield loss relationship. Notably, both of these parameters in this experiment were expressed post-flowering, and this may be a period when differential *P. medicaginis* inoculum production occurs among genotypes.

Both potential parameters indicative of inoculum production were PRR disease based. Findings for ranking similarities for the two genotypes at the base of the in-crop inoculum–yield loss and the proportion of symptomatic plants or early senescence-normalised yield loss relationships suggested simplistic relationships, whereby those genotypes with the least symptomatic plants or early senescence and yield loss will also provide lower inoculum development. If this is true, then yield loss experiments will need to be managed carefully to ensure that there is adequate disease pressure for foliage symptom development, as the two RIL disease phenotype experiments showed that differential inoculum production occurred under low disease RIL that did not develop foliage symptoms under dryland conditions and a low rainfall growing season. The differences in early senescence among chickpea genotypes may represent the effects of root disease damage contributing to premature foliage or crop maturity as shown in other pathosystems (Yang et al., 2016; Calamita et al., 2021). However, as shown for PRR of soybean where genotype maturity was evaluated for association with partial resistance (McBlain et al., 1991), chickpea genotype maturity may need to be considered as a contributing factor to the timing of senescence. If the priority is to identify RIL with low levels of *P. medicaginis* multiplication, then the evaluation of the proportion of symptomatic plants and area of early senescence as alternative parameters appears warranted.

In conclusion, we found some support for the hypothesis that *P. medicaginis* inoculum production differs among chickpea–*C. echinospermum* RIL and RIL parents with differing PRR resistance phenotypes. We found support for the hypothesis that inoculum production differs relative to normalised yield loss among RIL selected for low levels of PRR; however, comparisons were limited to a small set of genotypes due to variable inoculum measurements. We also found support for the evaluation of other parameters that were related to in-crop *P. medicaginis* inoculum production among chickpea genotypes.

## Data availability statement

The original contributions presented in the study are included in the article. Further inquiries can be directed to the corresponding author.

## Author contributions

SB, AD, DB, SH, and KH designed and conceived the study. SB and SH designed the experiments. SB and KH conducted the experiments. SB collected data and drafted the manuscript. All authors contributed to the article and approved the submitted version.
